# The State and Veterinary Science1Response to toast at the banquet of the graduating class of the New York College of Veterinary Surgeons.

**Published:** 1896-05

**Authors:** R. W. Hickman


### THE STATE AND VETERINARY SCIENCE.[1]

BY R. W. HICKMAN, V.M.D.

THE present relations of the State to veterinary science (and the public health, the two in so far as animal food at least is

[1] Response to toast at the banquet of the graduating class of the New York College of Veterinary Surgeons.

concerned being almost synonymous) are the outgrowth of industrial and economic interests, and exist even as they are to-day as the result of the law of self-preservation. This being the first law of nature is the one (which has, in degree) which will in a large measure act as a wholesome stimulus for a higher standard of attainment in veterinary education on the part of those engaged as well as those contemplating engaging in our noble profession.

Through enactments of various State legislatures competitive or civil-service examinations determine not only the fitness of applicants for appointment under the State government, but as to whether they are qualified to practise veterinary medicine at all within the borders of such State. Therefore the constantly increasing necessity for a more liberal, a broader, and more comprehensive education or enlightenment in every department of veterinary medical science. Frequent and disastrous outbreaks of disease in different States called for legislative enactments from an economical as well as a sanitary standpoint, and in order that such outbreaks of disease might be intelligently and profitably controlled or eradicated a staff of educated veterinarians was an inevitable necessity. Therefore, the economic and hygienic needs of the people designated the cause, while the effect has been a higher and broader plan of education in our colleges, and the result has already been decidedly salutary. I have too much sympathy for these graduates who have so patiently listened to me week after week and month after month, to burden you to-night with a lot of uninteresting data, and I imagine, judging from their examination papers, that they were not only awake but wide awake a greater part of the time. I will give you just a few salient points and only such data as may be necessary to show the beneficence of the relation of the State to veterinary science; how each is benefited by the other, or how cause and effect work together resulting in the greatest good to the people.

The first requisite in the control or eradication of disease is a knowledge of its nature and an ability to practically demonstrate such knowledge. The second requisite is legal authority to put such knowledge and ability into execution. This proposition (if accepted) establishes the relation without going further. But we have the evidence. Quite early in the nineteenth century cattle in Virginia were seized with a terribly fatal disease, the origin of which was traced to cattle that had been driven up from the South, and it was likewise noticed that the cattle from the South which communicated the disease were not affected themselves. This mystifying procedure after importations of Southern cattle into more Northern States and the West continued from time to time to be a source of considerable loss to owners of cattle as well as a source of great anxiety and agitation in the States suffering such losses. In 1868 Illinois, Indiana, and other States, notably our own, suffered immense losses through a large shipment of Southern cattle into the great Union Stockyards at Chicago, which were distributed over Illinois and Indiana, resulting in a widespread and fatal outbreak of disease in these States, and at the same time large numbers of cattle which had been exposed to the infection died in transit to or after arriving in the New York stockyards.

At this time the Metropolitan Board of Health and the New York State Cattle Commission, not understanding the exact nature of the malady or the possible dangers to the human family, made an effort to prevent or arrest the importation of diseased cattle from the West. During the same year some valuable investigations were made by Dr. R. C. Stiles for the Metropolitan Board of Health, and by Drs. John Gamgee, John S. Billings, and Curtice for the federal government. The most valuable contribution to the subject, however, from that time to the present was the determination of the boundary line of the permanently infected district by Dr. D. E. Salmon, Chief of the Bureau of Animal Industry, which bureau was organized with the present chief at its head in 1884, to whom we are likewise indebted for our knowledge of the manner in which the disease-germ (protozoon) is transmitted, so that, to-day, without interference with the great commercial cattle-interests of the South, their sale and transportation from below the quarantine line are conducted under regulations of the government bureau and under the supervision of federal inspectors to any State in the Union or at any season of the year, with no danger of disease so long as there is a proper observance of these regulations on the part of the railroad and other transportation companies. Through the efforts of the Bureau of Animal Industry in maintaining these regulations the dissemination of the disease has been successfully prevented, and where small outbreaks have occurred they were traceable to violations of the regulations by the railroad companies.

When, however, the magnitude of this industry is considered, the small number of cases is evidence both of the value of the determination of the quarantine line and of the efficiency of the bureau's work. As an illustration, let me tell you that I have seen in a single day over fifteen thousand Texas cattle arrive in the Union Stockyards, Chicago, everyone of which (though perfectly healthy) carrying on its body infection sufficient to infect a large herd of Northern cattle.

From February 15 to December 1, 1893, 64,184 carloads of cattle from the infected district were inspected by the inspectors of the bureau. These cars contained 1,737,380 head of cattle, which were placed in quarantine pens and kept separated from susceptible animals. This or even double the number can be handled with no resulting disease if there are no violations of the regulations by the transportation companies.

Dr. Salmon has shown that the Southern cattle-tick (*Bovphilus bovus*) is the carrier of the microörganism (*Pyrosoma bigeminum*) which causes Texas fever, and that an absence of ticks from Southern cattle or their trails entirely removes the danger of infection of Northern cattle.

Mr. Kleberg, Secretary of the Texas Live-stock Sanitary Commission, has claimed to be the inventor of a solution or bath and bath-tank, by means of which he can immerse 2000 head of cattle a day at a cost of one cent each and destroy all ticks on them without injury to the cattle. Numerous experiments have been tried at the department laboratory, but no chemical substance or compound has yet been discovered that can be used sufficiently concentrated to kill the ticks without producing great discomfort to the beasts or temporary injury to their skins. A bath that would do what Mr. Kleberg claims for his invention would remove the necessity for separating Southern from Northern cattle, as well as washing and disinfecting cars that had carried them North.

The history of contagious pleuro-pneumonia can be traced with comparative certainty back to the early part of the eighteenth century—1713 and 1714. From about 1769 we have frequent and well-authenticated accounts of its existence in various parts of Europe. During the period from 1790 to

1812 it was spread throughout a large portion of the Continent of Europe by cattle driven for the subsistence of the armies which marched and countermarched in all directions; existed in England in 1842; was introduced into the United States (Brooklyn, L. I.) in 1843; New Jersey in 1847; Massachusetts in 1859.

Massachusetts promptly commenced the work of eradication, and was successful during the period from 1860 to 1866. New York and New Jersey made an attempt at its extirpation in 1879, but failed. Previous to 1868 the disease had spread to the vicinity of Baltimore, and in 1883 was carried from there into Ohio through a purchase of Jersey cattle, and thus it was spread by the sale of cattle to Illinois, Missouri, and Kentucky in 1884 This widespread dissemination of such a dangerously contagious disease caused great alarm to the cattle-owners of the country, and when, in 1886, it was discovered in some of the large distillery stables about Chicago, and among cows pasturing on prairies in their vicinity, and reports were made of its existence in Pennsylvania, the necessity for active measures for its eradication was clearly apparent.

In 1887 Congress enlarged the appropriation available for this purpose, and gave more extended authority to the governmental department having the matter in charge, which, with the coöperation of the authorities of the several affected States, in a notably short time (characteristic of the snap and energy of the American) resulted in the complete extirpation of a plague that was menacing the great cattle-interests of our greatest of countries, the last case being destroyed in New York early in 1891 and in New Jersey in the spring of 1892. For a number of years we have had government quarantine stations, which are maintained under the regulations of the department, for the reception of cattle, sheep, and swine imported from foreign countries; thus we have and will prevent a reintroduction of the contagious pleuro-pneumonia, and have prevented the introduction of epizoötic eczema, which has been so prevalent in England and on the Continent, and possibly other veritable cattle-plagues.

But no legislation has been enacted providing for the inspection of horses imported into the United States, and we have already probably received from Europe one disease which has been known to exist there since 1796, and was doubtless introduced with some horses imported for breeding purposes. A number of animals were found to be infected in Illinois later, probably 200, 35 in Nebraska, and a few in South Dakota. These horses were all purchased and killed, and the expense to the government of eradicating this disease from the States named, including salaries, travelling expenses, and cost of horses, was $5232.54. The disease is known as dourine or *maladie du coit.* The course of the malady is slow. It first appears as a local affection, and becomes generalized after a few weeks or months. Animals may live for three or four years, being capable of disseminating the contagion during all this time.

The relations of the State in the broad, as well as the limited, sense to veterinary science is of great importance and considerable development, and is annually increasing in importance and magnitude far beyond the conception of those not directly interested, and, while at this time it would be inappropriate to go into all of the details, I feel that I would not meet the expectations of my colleagues if I failed to allude in particular to some of the more important branches of the work.

As to the scientific investigations of the bureau, these have been ably and conscientiously prosecuted with results of exceeding value. Hog-cholera and swine-plague have been distinctively described and separated, the etiology of each clearly demonstrated, and a course of treatment formulated and tested, giving promising results. Actinomycosis, formerly known by veterinarians as osteosarcoma, has likewise been carefully investigated, its cause, pathological anatomy, *and remedy*, clearly set forth; the latter by actual and numerous tests shown to be almost a specific for the cure of this disease. The diagnostic agents of tuberculosis and glanders—*i. e.*, tuberculin and mallein—have been largely distributed from the laboratory of the bureau to State authorities, State experiment stations, and educational institutions as a means of discovering the existence of tuberculosis in milch-cows and glanders in horses. Supplies of tuberculin have been sent to the proper authorities of thirty-three States; it is now being used at the rate of 2000 injections per month, with orders on file for 1000 injections per week from one State alone, and the publications of the bureau are the most valuable reference-books in the hands of the veterinary profession to-day, as a corps of veterinary inspectors is constantly engaged in field-work (making investigations of reported outbreaks of disease in different parts of the country, etc.), which, in addition to the inspection and post-mortem work at one hundred abattoirs and the different stockyards located in or near the large cities in various States, and the scientific investigations at the experiment stations and laboratories of the department, keep us informed of the most recent results of research in veterinary science.

